# Zinc Modulates Nanosilver-Induced Toxicity in Primary Neuronal Cultures

**DOI:** 10.1007/s12640-015-9583-3

**Published:** 2015-12-21

**Authors:** Elżbieta Ziemińska, Lidia Strużyńska

**Affiliations:** Laboratory of Pharmaconeurochemistry, Department of Neurochemistry, Mossakowski Medical Research Centre, Polish Academy of Sciences, 5 Pawińskiego str, 02-106 Warsaw, Poland; Laboratory of Pathoneurochemistry, Department of Neurochemistry, Mossakowski Medical Research Centre, Polish Academy of Sciences, 5 Pawińskiego str, 02-106 Warsaw, Poland

**Keywords:** Nanotoxicity, Zinc homeostasis, TPEN, Free radicals, NMDA receptor, Cell death

## Abstract

Silver nanoparticles (NAg) have recently become one of the most commonly used nanomaterials. Since the ability of nanosilver to enter the brain has been confirmed, there has been a need to investigate mechanisms of its neurotoxicity. We previously showed that primary neuronal cultures treated with nanosilver undergo destabilization of calcium homeostasis via a mechanism involving glutamatergic NMDA receptors. Considering the fact that zinc interacts with these receptors, the aim of the present study was to examine the role of zinc in mechanisms of neuronal cell death in primary cultures. In cells treated with nanosilver, we noted an imbalance between extracellular and intracellular zinc levels. Thus, the influence of zinc deficiency and supplementation on nanosilver-evoked cytotoxicity was investigated by treatment with TPEN (a chelator of zinc ions), or ZnCl_2_, respectively. Elimination of zinc leads to complete death of nanosilver-treated CGCs. In contrast, supplementation with ZnCl_2_ increases viability of CGCs in a dose-dependent manner. Addition of zinc provided protection against the extra/intracellular calcium imbalance in a manner similar to MK-801, an antagonist of NMDA receptors. Zinc chelation by TPEN decreases the mitochondrial potential and dramatically increases the rate of production of reactive oxygen species. Our results indicate that zinc supplementation positively influences nanosilver-evoked changes in CGCs. This is presumed to be due to an inhibitory effect on NMDA-sensitive calcium channels.

## Introduction

In recent years, silver nanoparticles (NAg) have become an extremely popular nanotechnology product widely used in many applications. Their strong antibacterial potential is useful in medical products (Samuel and Gugenbichler 2004; Zheng et al., 2010) and they are also found in a growth market of consumer articles (for review see: Schluesener and Schluesener 2013). Thus, as the risk of increased human exposure appears, further information on cellular metabolic pathways affected by nanosilver is needed.

Cytotoxicity of nanosilver has been confirmed in both in vitro and in vivo studies (Haase et al. 2012; Strużyński et al. 2014; Yin et al. 2013) indicating that the induction of oxidative stress underlies nanosilver-induced cell death. It has been reported that nanosilver has an influence on mitochondrial function, generation of free radicals (AshaRani et al. 2009; Hussain et al. 2005; Singh and Ramarao 2012) and induction of apoptosis due to lowering of the cellular antioxidant glutathione (Piao et al. 2011). The high reactivity of NAg, resulting in enhanced ability to cross cell membranes, is of great concern in neuroscience due to the progressive development of theragnosis, a new concept in next-generation medicine that combines simultaneous diagnosis and therapy with the use of nanoparticles, including NAg (Leite et al. 2015). Since NAg have been shown to enter the brain by permeating the blood–brain barrier (BBB) (Hoet et al. 2004; Sharma et al. 2009) and significantly accumulate in this organ, it is important to clarified their neurotoxic effects.

We have previously investigated the mechanisms of cell death in cultured cerebellar granule cells (CGC) exposed to silver nanoparticles (Ziemińska et al. 2014). The results of this study indicated that overactivation of glutamatergic *N*-methyl-d-aspartate receptors (NMDARs) is involved in neuronal cell death via excessive entry of calcium ions followed by an intracellular calcium imbalance. However, it has been shown that in addition to calcium, zinc (Zn) may have a negative effect on cell function in a process which leads to cell death (Lozier et al. 2012). In glutamatergic neurons, zinc is largely co-localized with the excitatory neurotransmitter glutamate (Frederickson 1989) and is synaptically released upon cell activation (Assaf and Chung 1984). If so, under the excitotoxic conditions produced by overactivation of glutamatergic receptors, it is probable that excessive levels of extracellular zinc exist. In view of the data indicating that Zn may modify voltage- and ligand-gated channels (Harrison and Gibson 1994), and especially NMDA receptor channels (Peters et al. 1987), we suspect that zinc is playing an important role in the pathological mechanisms operating during nanosilver-mediated toxicity toward CGCs in our experimental design.

Zinc plays an important role in a wide variety of cellular processes as a cofactor of more than 300 enzymes involved in a majority of metabolic processes (Beyersmann and Haase 2001). In neuronal cells, deregulation of zinc homeostasis may occur as a consequence of an imbalance between synaptic and intracellular pools of this metal. It has been shown that this may contribute to neuronal cell death in numerous pathologies of the CNS, including ischemia and seizures (Koh et al. 1996; Tonder et al. 1990).

It is known that zinc has a dual effect in cell, neurotoxic, or neuroprotective, although mechanisms of its action are not precisely known (Sobieszczańska et al. 2012), particularly under pathological conditions. Intracellular zinc is bound to the cell membranes and cytoplasmic proteins (structural zinc) or is present in the cytoplasm as so called “free zinc” (Vallee and Falchuk 1993). In the CNS, zinc is also present at relatively high levels pooled within synaptic vesicles together with glutamate in glutamatergic neurons.

There are several mechanisms maintaining cellular zinc homeostasis. These mechanisms involve metallothioneins (Aschner et al. 1997) and specific transporters (Palmiter and Findley 1995). Transmembrane transporter Zn-T1 exports zinc ions out of the cell; Zn-T2 and Zn-T3 are, respectively, responsible for transporting zinc ions from the cytoplasm to endosomes and synaptic vesicles. The proper zinc gradient (which is lower inside than outside the cell) is provided by energy-dependent process. Thus, under conditions leading to decreased ATP production, the intracellular zinc concentration increases. This also occurs in response to oxidative stress resulting from the release of zinc from metallothioneins (Aizmenman et al. 2000). Other processes leading to enhanced release of zinc from glutamatergic nerve endings may result in zinc entering the cell through voltage-gated calcium channels (Kim et al. 2000) or AMPA/KA glutamate receptors (Sensi et al. 1997). An excess of intracellular zinc disturbs oxygen metabolism by inhibiting mitochondrial electron transport, disrupts calcium homeostasis, and overactivates calcium-mediated enzymes leading finally to cell death (Trombley et al. 2000; Lobner et al. 2000).

Thus, in the present paper, we investigate further mechanisms of cell death under nanosilver toxicity conditions, with particular attention to the role of zinc ions. Cerebellar granule cells (CGCs) cultured in the presence of nanosilver are studied under conditions of zinc deficiency and increased levels of zinc.

## Materials and Methods

### Materials

Fluo-3AM, FluoZin-1, FluoZin-3AM, 6-Carboxy-2′,7′-dichlorofluorescein diacetate (DCF), and rhodamine 123 (R123) were obtained from Molecular Probes (Eugene, OR, USA). ^45^CaCl_2_ was produced by Polatom Sp. z o.o., Otwock—Swierk, Poland. Other chemicals, including nanosilver and cell culture materials were purchased from Sigma-Aldrich Chemical Co. (St. Louis, MO, USA). All reagents were of analytical grade.

### Preparation of Silver Nanoparticles (NAg)

Commercially available nanosilver was used in the present study. Nanosilver is defined by the manufacturer (Sigma-Aldrich) as a mixture of polyvinylpyrrolidone-coated nanoparticles (0.2 % PVP-coated nanosilver) less than 100 nm in diameter. We previously characterized and investigated nanosilver produced by this manufacturer in toxicological studies (Strużyńska et al. 2014; Zieminska et al. 2014). However, for the purpose of the present work, characterization of the degree of dispersion and particle size distribution was performed using transmission electron microscopic analysis (JEM-1200EX, Jeol) both in stock solution with fetal calf serum (FCS) and in exposure media (BME, Locke buffer).

A stock solution of nanosilver in fetal calf serum was freshly prepared just prior to its addition to the CGC culture (2 × 10^6^/well) in order to avoid the release of Ag^+^ ions from the particle surface (Kittler et al. 2010). Stock solution was sonicated in proper conditions (6 × 20 s, 35 W, BANDELIN sonoplus HD 70) to prevent sedimentation and agglomeration of nanoparticles as well as hitting of the solution and denaturation of serum proteins. Additionally, stock solution was filtered using a 0.22-µM microfilter.

The same volume of supernatant (obtained after centrifugation of above-described stock solution of NAg at 15,000×*g*, 30 min), containing Ag^+^ liberated from the nanoparticle surface, was used as a control to define the effects characteristic for ionic or “nano” form of silver. Absence of nanoparticles in supernatant was confirmed using TEM.

We also check whether NAg, under absence of biological material, interact with fluorescent dyes used in further experiments and we noticed any interference.

### Cell Culture and Experimental Design of Nanosilver Cytotoxicity

Primary cultures of cerebellar granule cells (CGCs) were prepared from 7-day-old Wistar rats of both sexes as described previously (Ziemińska et al. 2010, 2014). Procedures using rat pups were performed in accordance with international standards of animal care guidelines and were approved by the Local Care of Experimental Animals Committee. Briefly, after decapitation, cerebella were rapidly removed, cut into 400 μm cubes, and subjected to trypsinization. After centrifugation, the cells were suspended in basal Eagle’s medium (BME) and seeded on plates (NUNC) coated with poly-l-lysine, at a densities of 4 × 10^6^, 2 × 10^6^ or 1 × 10^6^ cells per well or 15 × 10^6^ per flash. In each case, the cell suspension was supplemented with 10 % fetal calf serum, 25 mM KCl, 4 mM glutamine, streptomycin (50 μg/mL), and penicillin (50 U/mL). The CGCs were used in experiments after 7 days.

On the 7th day of culture, serum solutions of nanosilver (NAg)/supernatant (Ag^+^) were added directly to BME growth medium to obtain final concentrations in the range of 2.5–10 or 75 µg/mL. In most experiments, nanosilver in concentration of 75 µg/mL (NAg 75) and supernatant obtained from this solution (super 75) were applied. In serum, nanoparticles sediment very slowly and this characteristic provides reproducibility during addition to the cultures. However, since serum contains trace amounts of its own glutamate, calcium, and zinc ions, it is also necessary to compare the results obtained for NAg with control culture supplemented with the volume of serum equal to the volume of NAg or supernatant added to the experimental system (control + serum 75). The final concentration of FCS in this experimental design was 10.75 %. Apart from this, untreated cells were also used as a negative control.

Additionally, the culture medium contained 20 μM of the selective zinc ions chelator TPEN (20 μM *N*,*N*,*N*,*N*-tetrakis(2-pirydylmethyl) ethylenediamine), 10–50 μM ZnCl_2_ or 0.5 µM of MK-801 (an antagonist of glutamatergic NMDA receptors), or 10–50 µM CNQX (an antagonist of AMPA/KA glutamatergic receptors). The cells were cultured for 24 h and then the viability of neurons was assessed by staining with 0.5 µg/ml propidium iodide (PI) or with the fluorescent dyes calcein and EtHD. PI is toxic for the neurons and stains only dead cells, so prior to staining with PI, the cells were fixed with 80 % methanol. Cells that were alive in the time of fixation differed morphologically from dead ones. Fluorescence microscopy was employed to count living and dead neurons (Zeiss-Axiovert, Germany) as described previously (Ziemińska et al. 2010, 2014). Results were expressed as percentage of live cells in proportion to all cells.

To visualize changes in nuclear chromatin, LSM 510 confocal microscope (Carl Zeiss AG, Germany) was used. Images of fixed and PI-stained cells were recorded using 546 nm HeNe laser for excitation of fluorescence. The emission was measure at *λ* 617 nm. Studies using confocal microscopy were performed in Laboratory of Advanced Techniques, Mossakowski Medical Research Centre, Polish Academy of Sciences.

### Measurement of Intracellular Zinc Levels with Fluorescent Dye FluoZin-3AM

Changes in the intracellular Zn^2+^ concentration were measured fluorometrically. The cells (1 × 10^6^/well) were incubated at 37 °C for 60 min in growth medium with 3 μM FluoZin-3AM. Then, the cells were twice washed with Locke 5 medium and examined using confocal microscope (LSM 510, Carl Zeiss AG, Germany) after addition of NAg/Ag^+^. The fluorescence of FluoZin-3 induced by argon laser at 488 nm was measured at 530 nm every 30 s during 15 min. The results are presented as percentage changes in the intensity of fluorescence in relation to its basal level (*F*/*F*_o_). The data were obtained from three independent trials using separate CGC cultures and presented as means of 15 randomly selected objects.

### Measurement of Zinc Levels with Fluorescent Dyes FluoZin-1

CGCs (15 × 10^6^/bottle) were transferred to Locke 25 medium and incubated 30 min with 75 µg/mL NAg or Ag^+^ in the presence of 0.75 % FCS. Then, to avoid interference with extracellular zinc from serum, the cells were three times washed with fresh Locke medium containing 154 mM NaCl, 5 mM KCl, 4 mM NaHCO_3_, 2.3 mM CaCl_2_, 5 mM HEPES (pH 7.4), and 5 mM glucose. Since the probe does not penetrate the membrane, the cells from each bottle were lysed in 0.5 M NaOH to provide visualization of zinc in the intracellular space. Then 100 μL of solution was incubated with 1 μM FluoZin-1 and fluorescence was measured using a microplate reader (FLUOstar Omega, Germany) at 485 nm excitation and 538 nm emission wavelengths. Addition of NaOH alone had no effect on the readout of fluorescence (control with Locke solution with ZnCl_2_ versus the same concentration of ZnCl_2_ in NaOH).

### Uptake of Radioactive Calcium

The cells (4 × 10^6^/well) were pre-incubated at 37 °C for 10 min in Locke 5 medium. Radioactive calcium (1 μCi/well) was added together with 75 μg/mL NAg/Ag^+^ or other substances and after 10 min incubation at 37 °C, the cells were washed 3× with ice-cold glucose and calcium-free medium containing 2 mM EGTA. After lysing the cells in 0.5 M NaOH, radioactive calcium uptake was measured using a Wallac 1409 liquid scintillation counter (Wallac, Turku, Finland).

### Loading of Cells with fluo-3 AM and Fluorescence Measurements

CGCs (1 × 10^6^/well) were loaded with the fluorescent calcium-sensitive probe, 4 μM fluo-3 AM, at 37 °C for 30 min. The cells were washed with the Locke 5 buffer to terminate loading. Changes in fluorescence after addition of all tested compounds were recorded at 1-min intervals over a 30-min period, using a microplate reader (FLUOstar Omega, Germany) at 485-nm excitation and 538-nm emission wavelengths.

### Measurement of Mitochondrial Membrane Potential in CGC

Rhodamine123 (R123) was added to the cultures (1 × 10^6^/well) to a final concentration of 10 μM for 30 min at 37 °C. Decrease in mitochondrial membrane potential was monitored by increased intracellular fluorescence. After preincubation with R123, cells were washed with Locke 5 buffer and treated with 75 µg/mL NAg/Ag^+^ alone or mixed with other tested compounds. Changes of fluorescence were recorded every 3 min, over a 1-h period, using a microplate reader (Fluoroscan, LabScan, Finland) at 485-nm excitation and 538-nm emission wavelengths.

### Measurement of Free Radicals Production in CGC

The fluorescent dye 2′,7′-dichlorofluorescin diacetate (DCF) was added to the cultures to a final concentration of 100 μM and used to evaluate free radical production. After a 30-min incubation period, cells (1 × 10^6^/well) were washed with Locke 5 buffer and treated with 75 µg/mL nanosilver, supernatant alone, or in combination with other tested compounds. Changes in fluorescence were recorded at 1 min intervals, over a 30-min incubation period, using a microplate reader (FLUOstar Omega, Germany) at 485-nm excitation and 538-nm emission wavelengths.

### Statistical Analysis

Results are expressed as mean ± SD of the number of cultures indicated below each figure. Inter-group comparisons were made using the one-way analysis of variance (ANOVA) with Bonferroni correction for multiple comparisons. The significance level was set as *P* < 0.05.

## Results

### Silver Nanoparticles

Commercially available 0.2 % polyvinylpyrrolidone (PVP)-coated nanosilver particles <100 nm were used in the experiments. TEM studies revealed that the nanoparticles generally have a spherical shape and do not form aggregates in the stock solution either in the presence of serum or culture media (BME and Locke buffer). The size distribution estimation indicated that the vast majority (88 %) of nanosilver particles were between 5 and 25 nm in diameter and more than half (65 %) did not exceed 10 nm in diameter (Table 1). No nanoparticles were detected in the supernatant obtained after centrifugation of the stock solution of silver nanoparticles.

**Table 1:** The size distribution of silver nanoparticles in serum stock solution

Size (nm)	%
5	42
10	23
15	13
20	10
25	7
30	3
35	2

The nanoparticles of varying sizes were prepared by the procedure described in “Materials and Methods”. Calculations were made using 15 TEM micrographs and expressed as a percentage

### Changes in the Level of Zinc in CGCs Exposed to Nanosilver Particles

We examined whether incubation of cells with NAg/Ag^+^ influences the level of total intracellular zinc and its cytosolic “free” pool. To assess cytosolic zinc changes, we used cell-permeable fluorescent dye FluoZin-3AM, a Zn^2+^-selective indicator, which exhibits high Zn^2+^-binding affinity unperturbed by Ca^2+^ concentrations up to at least 1 µM. In addition, this dye exhibits a >50-fold increase in fluorescence in response to saturating levels of Zn^2+^.

**Fig. 1:**
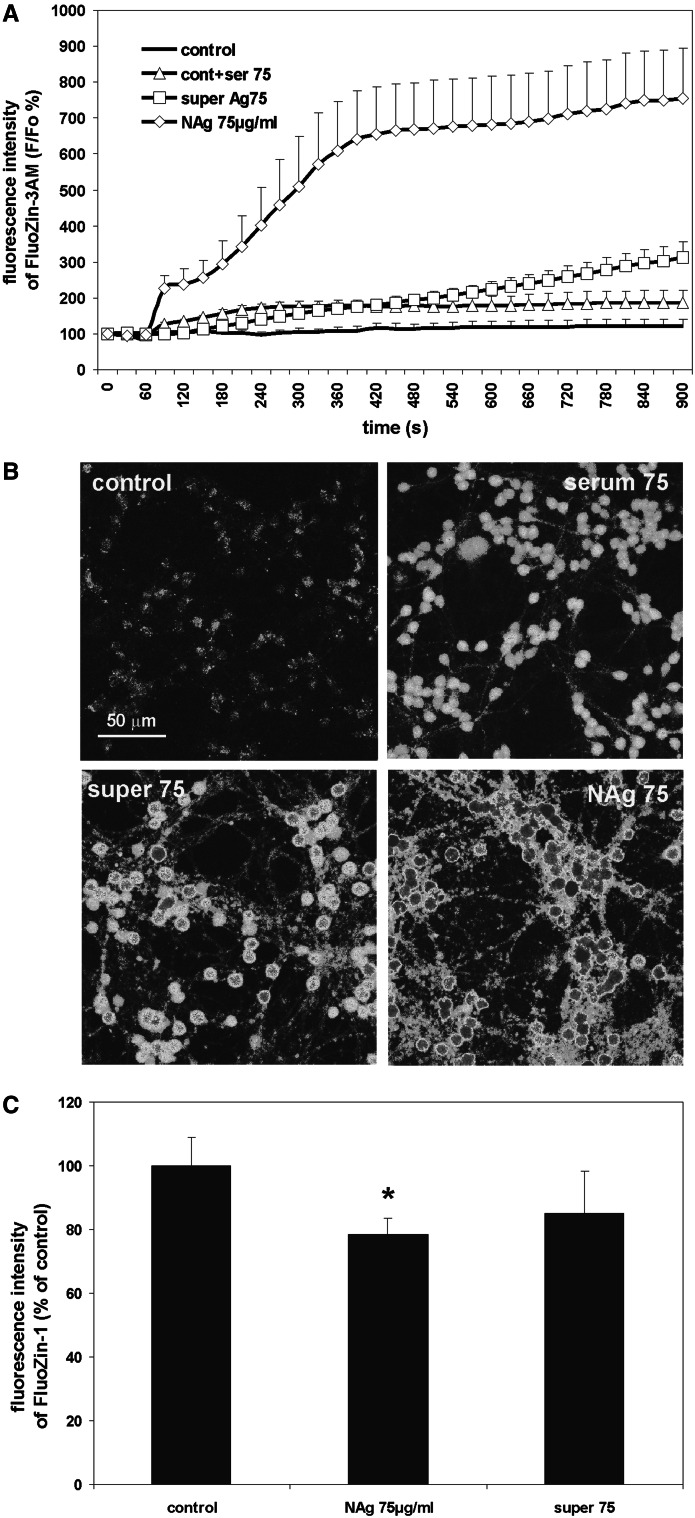
The influence of nanosilver and silver ions on intra- and extracellular zinc levels: **a** cytosolic zinc-derived fluorescence intensity measured using FluoZin-3AM in Locke medium 60 s after adding 75 µg/mL of nanosilver (NAg 75 µg/mL), supernatant with ionic silver obtained from NAg solution (super 75) or the volume of serum equal to the volume of NAg, or supernatant added to the experimental system (control + serum 75). Changes in fluorescence intensity are presented in relation to its basal level (F/Fo). The results are mean ± SD obtained from three independent trials using separate CGC cultures and presented as means of 15 randomly selected objects. **b** Representative images taken in the 870th sec showing changes in fluorescence intensity in cell cultures subjected to the conditions described in (**a**). **c** zinc-derived fluorescence intensity measured using the fluorescent dye FluoZin-1 in cell lysates (15 × 10^6^) after 30 min of incubation with 75 µg/mL of nanosilver (NAg 75 µg/mL) or ionic silver (super 75) prepared as described in “Materials and Methods.” The results are mean ± SD from 6 measurements obtained from three independent cell cultures and presented as % of the fluorescence intensity in control cultures. *P < 0.05 versus negative control (one-way ANOVA)

As it is seen (Fig. 1a, b), addition of 75 µg/ml NAg results in a rapid (during 60 s) and a very high (sevenfold) increase in cytosolic zinc level compared to its basal level in control untreated cells (max. time of signal recording 15 min). In parallel, fluorescence was recorded in medium around the cells (Fig. 1b). It was significantly less pronounced after application of supernatant and did not occur when only serum was added. Since FluoZin-3 bounds only free zinc, we claimed that cytosolic pool of free zinc increases rapidly after NAg treatment and is subsequently partially released from the cell to the culture medium.

We also used fluorescent dye FluoZin-1 to measure the fluorescence intensity in cell lysates (15 × 10^6^) over a longer period of time (after 30 min). FluoZin-1 is a cell-impermeable indicator designed to detect zinc concentrations in the 0–100 µM range with minimal interference with calcium sensitivity. Decreased fluorescence in cell lysates reflecting decrease in the intracellular zinc (total zinc i.e., free cytosolic and sequestered in cellular organelles) was noted after exposure to both NAg and Ag^+^. However, destabilization of cellular zinc equilibrium was significantly more evident after exposure to nanosilver particles and reached about 20 % (*P < 0.05 versus control) (Fig. 1c).

### Viability of Nanosilver-Treated CGC in the Presence or Absence of Zinc

We have previously checked that application of NAg or supernatant (Ag^+^) in the concentration range of 2.5–25 µg/mL exhibited no visible effect on the viability of neurons after 24 h of exposure (Ziemińska et al. 2014). The statistically significant decrease in cells viability occurred after addition of NAg in the concentrations ≥50 µg/mL. The number of living cells declined in a dose-dependent manner from 60 to 30 % of control value.

Results after addition of respective amounts of supernatant (Ag^+^) were generally comparable, but slightly greater in numbers of cells survived (55–70 %) relative to respective doses of NAg. Thus, in the current experiments, we used the concentration of silver nanoparticles in the presence of which viability of neurons decreased to about 50 % of control (75 µg/mL). Such marked dose–response effect allowed us to test modulatory effects of investigated substances on mechanisms leading to cell death.

**Fig. 2:**
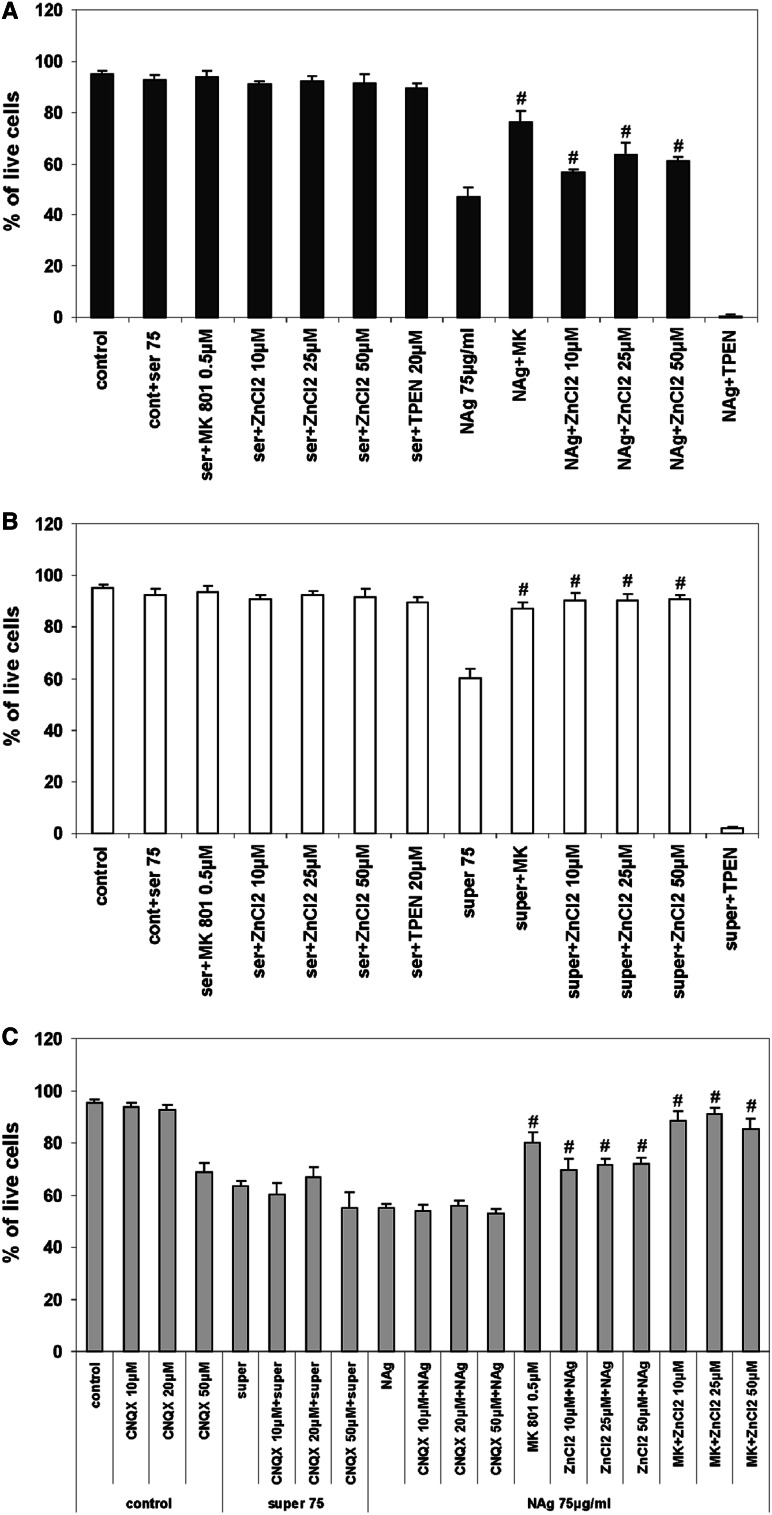
The influence of TPEN, ZnCl_2_, MK-801, and CNQX on viability of CGCs presented as a percentage of live cells in cultures exposed for 24 h to 75 µg/mL of nanosilver (NAg 75 µg/mL) or ionic silver (super 75) prepared as described in “Materials and Methods.” The death effect of TPEN and the protective effect of ZnCl_2_ and MK-801 in **a** NAg-exposed cells, **b** ionic silver-exposed cells (super 75), **c** synergistic effect of ZnCl_2_ and MK-801 and the lack of CNQX effect in NAg-exposed cultures. The results are presented as mean ± SD from four independent experiments. ^#^ *P* < 0.05 and ^##^ *P* < 0.01 versus NAg/super 75-treated cells (one-way ANOVA)

Cell viability was assessed by propidium iodide (PI) or with the fluorescent dyes calcein and EtHD 24 h after addition of nanosilver particles (Fig. 2a) or supernatant (Ag^+^) (Fig. 2b). In the presence of either NAg or Ag^+^ at a concentration of 75 µg/mL, there was an apparent decrease in the number of living cells (47 and 60 % of the control value, respectively). Zinc chelation with 20 µM TPEN strongly aggravated cell death induced by NAg and Ag^+^. Addition of zinc to the cultured neurons in the concentration range of 10–50 µM increased the viability of the cells in a dose-dependent manner. In nanosilver-treated cells, the positive effect of zinc was less evident and did not exceed 20 %, whereas in Ag^+^-treated cells it was more pronounced in over 90 % of cells. Application of a noncompetitive antagonist of NMDA receptors (MK-801) significantly increased the number of living cells in nanosilver-treated cultures, exceeding the positive effect of zinc, by about 13 %. In Ag^+^-treated cultures, MK-801 gave neuroprotection at the same level as zinc. Positive effect of MK-801 was observed only in this type of experiments, under long-lasting exposure to Ag^+^ (24 h). In contrast to nanosilver-treated cells, further experiments performed on cells exposed for 30–60 min to Ag^+^ did not reveal positive effect of NMDAR antagonist.

In NAg-exposed cultures, cell viability after simultaneous addition of MK-801 and ZnCl_2_ to the growth medium was also assessed in order to observe potential synergistic effect. Combined effect of both neuroprotectants exceeded that derived from MK-801, increasing % of living neurons almost to the control value (91.5 vs. 95 %). However, that tendency was statistically insignificant (Fig. 2c). Since involvement of other ionotropic glutamate receptors into NAg-induced excitotoxic cell death is not excluded, we tested participation of AMPA/KA receptors in this mechanism. Receptors’ antagonist CNQX was added in concentrations 10–50 µM before application of NAg or Ag^+^, but in any case, we did not notice neuroprotective effect (Fig. 2c).

**Fig. 3:**
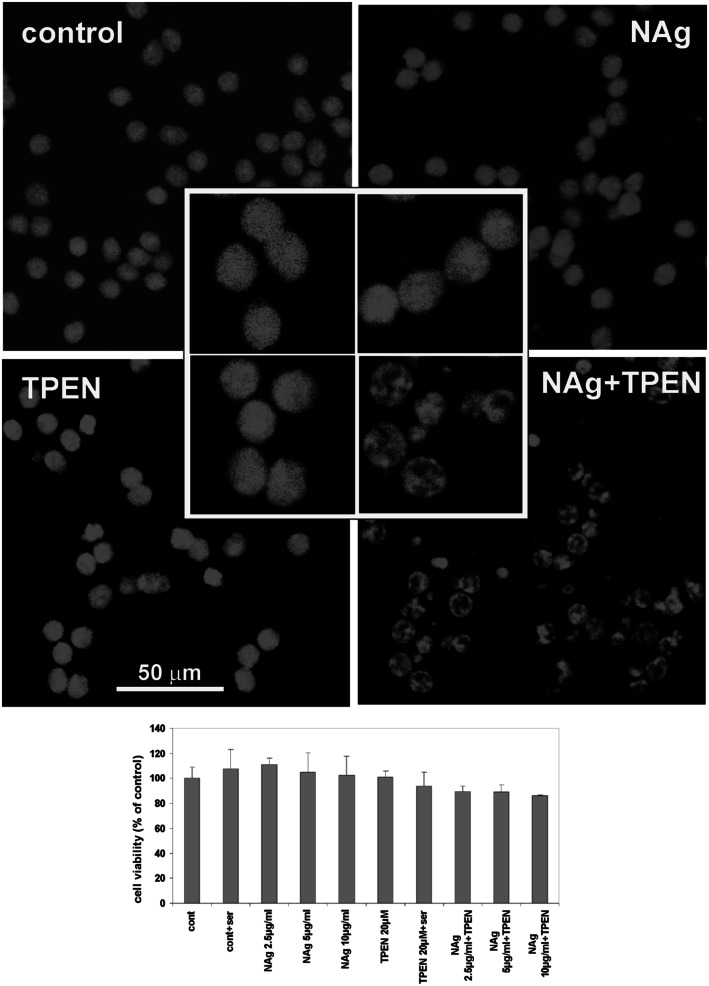
Images of cell nuclei fixed with methanol and stained with propidium iodide in control CGC cultures and in cultures treated with 20 μM TPEN, 10 μg/mL nanosilver particles (NAg) or both (NAg + TPEN). Chromatin changes are visible in cells exposed concomitantly to nanosilver and TPEN. The *graph* shows the results of tests for cell viability performed simultaneously with the fluorescent dies calcein and ethidium homodimer (EtHD). Although a tendency toward decreased viability of cells treated with 2.5–10 µg/mL nanosilver and 20 µM TPEN was observed, it was found to be statistically insignificant, *P* > 0.05 (one-way ANOVA). Data are presented as mean ± SD from six independent experiments. Neurons displayed characteristics of living cells despite obvious morphological changes in the nuclei. Inserts are magnified 2×

Further we observed neurons after a 24-h incubation period with low concentrations of NAg/Ag^+^ (2.5–10 μg/mL) and TPEN to investigate the effect of zinc deficiency. As we stated before, applied concentrations of nanosilver particles did not result in cell death (Ziemińska et al. 2014). However, parallel chelation of zinc influenced the appearance of nuclei in the cells (Fig. 3). Confocal images of cells visualized with propidium iodide exhibited changes in chromatin which may indicate induction of processes leading to their condensation, resembling the early changes of apoptotic nature. However, simultaneously performed tests of viability using calcein and ethidium homodimer (EtHD) showed that despite these morphological changes, the cells remain alive (Fig. 3, graph). In the case of Ag^+^-treated cells, we did not observe a similar reaction in the presence of TPEN.

### Influence of Zinc on Cellular Calcium Levels and Calcium Uptake in CGCs Exposed to Nanosilver

#### ^45^Ca Uptake

**Fig. 4:**
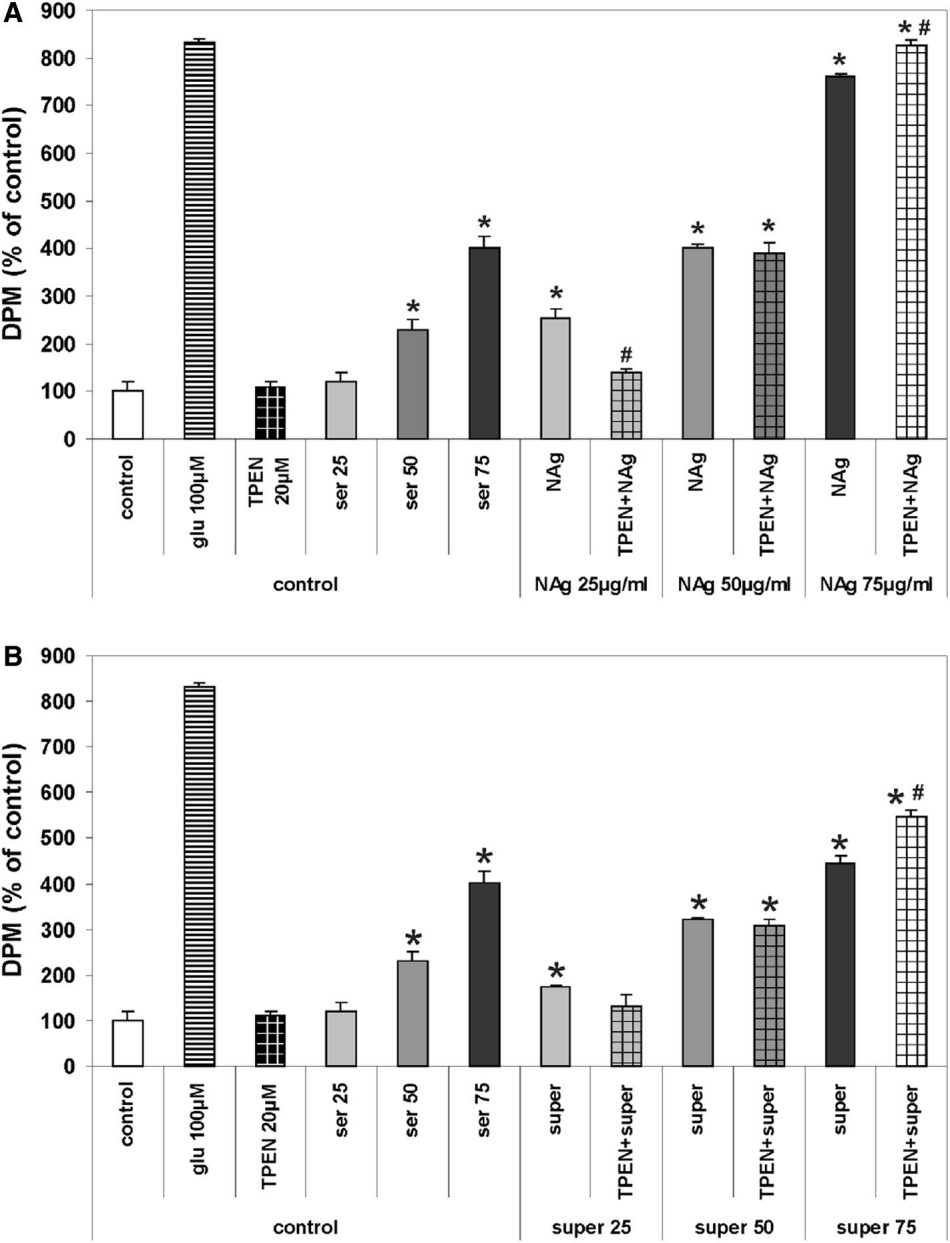
The effect of the zinc chelator TPEN on the uptake of radioactive calcium by CGCs exposed to different concentrations of **a** nanosilver particles (25–75 µg/mL) or **b** ionic silver (super 25–75) for 10 min. Glutamate (glu 100 µM), an agonist of NMDARs, was used as a positive control. Cultures of CGC supplemented with serum were used as a control of the serum effect (control + ser). Accumulated ^45^Ca was measured as nuclide disintegrations per minute (DPM) and expressed as a percentage of untreated control. The results are presented as mean ± SD from four independent experiments. *P < 0.05 versus control untreated cells; ^#^ *P* < 0.05 versus NAg-treated cells (one-way ANOVA)

The treatment of cultures with nanosilver within the concentration range of 25–75 μg/mL increases calcium uptake by CGCs in a dose-dependent manner. The amount of calcium taken up by cells was found to be maximal in the presence of 75 μg/mL nanosilver and almost equal to the amount taken up during stimulation by 100 μM glutamate which acts as an NMDAR agonist (the positive control in our experimental design) (Fig. 4a). TPEN added to neurons treated simultaneously with 25 µg/mL nanosilver significantly decreased the rate of uptake of radioactive calcium by 110 % (*P < 0.05 vs. NAg). TPEN added to neurons treated with 50 μg/mL nanosilver did not influence the calcium uptake. In the presence of the highest used concentration of nanosilver (75 μg/mL), TPEN stimulated calcium uptake to a level similar to that attained after addition of 100 μM glutamate i.e., 750 % over control value; *P < 0.05 and 66 % over NAg alone (Fig. 4a). Calcium uptake in CGCs incubated for 10 min with Ag^+^ (super 75) increased relative to control untreated cells but was lower by about 310 % compared to CGCs treated with 75 μg/mL nanosilver TPEN exerted visible effect only when added together with the highest concentration of Ag^+^ (super 75), resulting in a slight increase in the rate of Ca uptake (Fig. 4b).

**Fig. 5:**
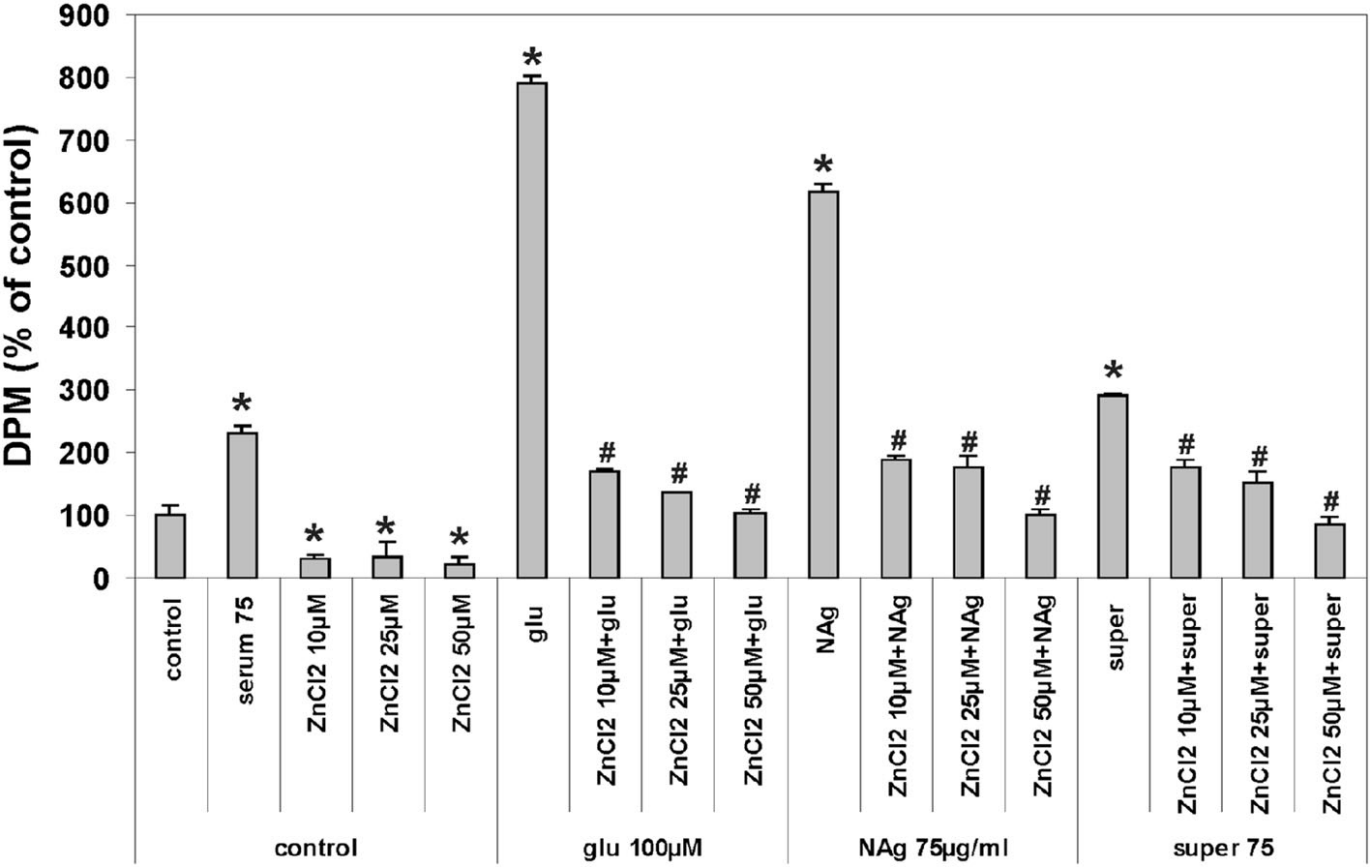
The effect of zinc supplementation (10, 25 and 50 µM ZnCl_2_) on the uptake of radioactive calcium by CGCs exposed to 75 µg/mL nanosilver (NAg) or ionic silver (super 75) for 10 min. Glutamate (glu 100 µM), an agonist of NMDARs, was used as a positive control. Cultures of CGC supplemented with serum were used as a control of the serum effect (serum 75). Accumulated ^45^Ca was measured as nuclide disintegrations per minute (DPM) and expressed as a percentage of untreated control. The results are presented as mean ± SD from four independent experiments. *P < 0.05 versus control untreated cells. ^#^ *P* < 0.05 versus NAg/super 75-treated cells (one-way ANOVA)

Additional supplementation of the culture medium with 10–50 μM ZnCl_2_ resulted in strong and dose-dependent inhibition of calcium uptake in glutamate- or nanosilver-treated cultures (about 700 and 500 %, respectively), so as in control cells. In Ag^+^-exposed cultures, the effect was less pronounced (about 200 %) (Fig. 5).

#### Intracellular Calcium Level

The intracellular calcium level which reflects uptake from the extracellular space and release from intracellular stores was monitored using the fluorescent dye fluo3-AM. According to the available data, virtually all fluorescent calcium probes also bind zinc ions. Changes in the fluorescence signal of these probes often reflect the simultaneously occurring changes in the concentrations of both calcium and zinc (Dineley 2007).

Previously we found that there is a 200 % increase in the intracellular calcium level over the control level in CGCs after a 30-min incubation with 75 µg/mL nanosilver (Ziemińska et al. 2014). Moreover, we observed that the total fluo3-AM fluorescence signal recorded in neuronal cytosol after nanosilver exposure is a mixed calcium-zinc signal. The zinc-derived fluorescent component was found to be unstable, dependent upon nanosilver concentration and changes in the range of 6–35 % with addition of 10 μg/mL (and less) or 75 μg/mL nanosilver, respectively.

**Fig. 6:**
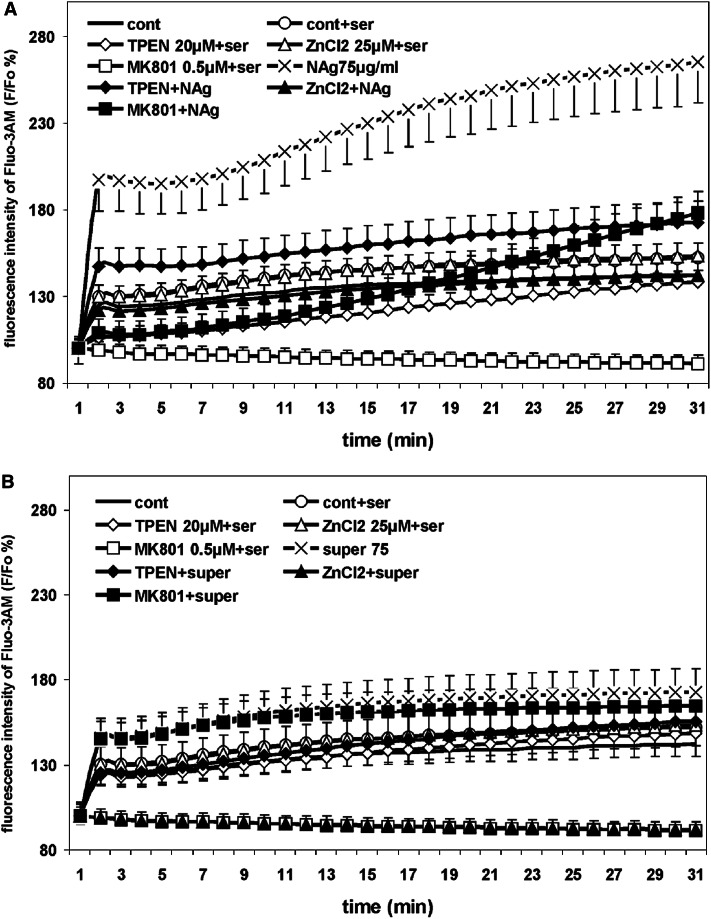
The influence of TPEN, ZnCl_2_ and MK-801 on intracellular Ca^2+^/Zn^2+^ levels in CGCs cultured in the presence of 75 µg/mL nanosilver (NAg) (**a**) or ionic silver (super 75) (**b**). Fluorescence intensity was measured using fluo-3AM and expressed as a percentage of the basal level (fluo-3AM F/Fo). The results are presented as mean ± SD from four independent experiments

These results confirm that in the presence of 75 μg/mL nanosilver, the intracellular calcium/zinc levels (as measured by fluorescence) increase significantly (*P < 0.05) by about 120 % relative to control cultures (Fig. 6a). Zinc chelation by TPEN significantly decreases the Ca/Zn signal (*P < 0.05). Addition of MK-801 also reduces the level of fluorescence, although this effect was found to be unstable during the incubation time. The most pronounced and time-stable blockage of the increase in intracellular Ca^2+^/Zn^2+^ was observed after addition of 25 μM ZnCl_2_. The recorded signal decreased to the level of the control.

In the Ag^+^-exposed cultures, we did not observe such a significant increase in intracellular Ca^2+/^Zn^2+^ levels or reduction thereof by MK-801 (Fig. 6b). However, in the presence of ZnCl_2_^+^, the fluorescence signal of Ca^2+^/Zn^2^ was lower than in the control untreated cultures.

### Influence of Zinc on Mitochondrial Potential and ROS Production Under Nanosilver Toxicity Conditions

Changes in the mitochondrial potential of CGCs were monitored using the fluorescent marker R123. Increased fluorescence reflects a drop in the mitochondrial membrane potential.

**Fig. 7:**
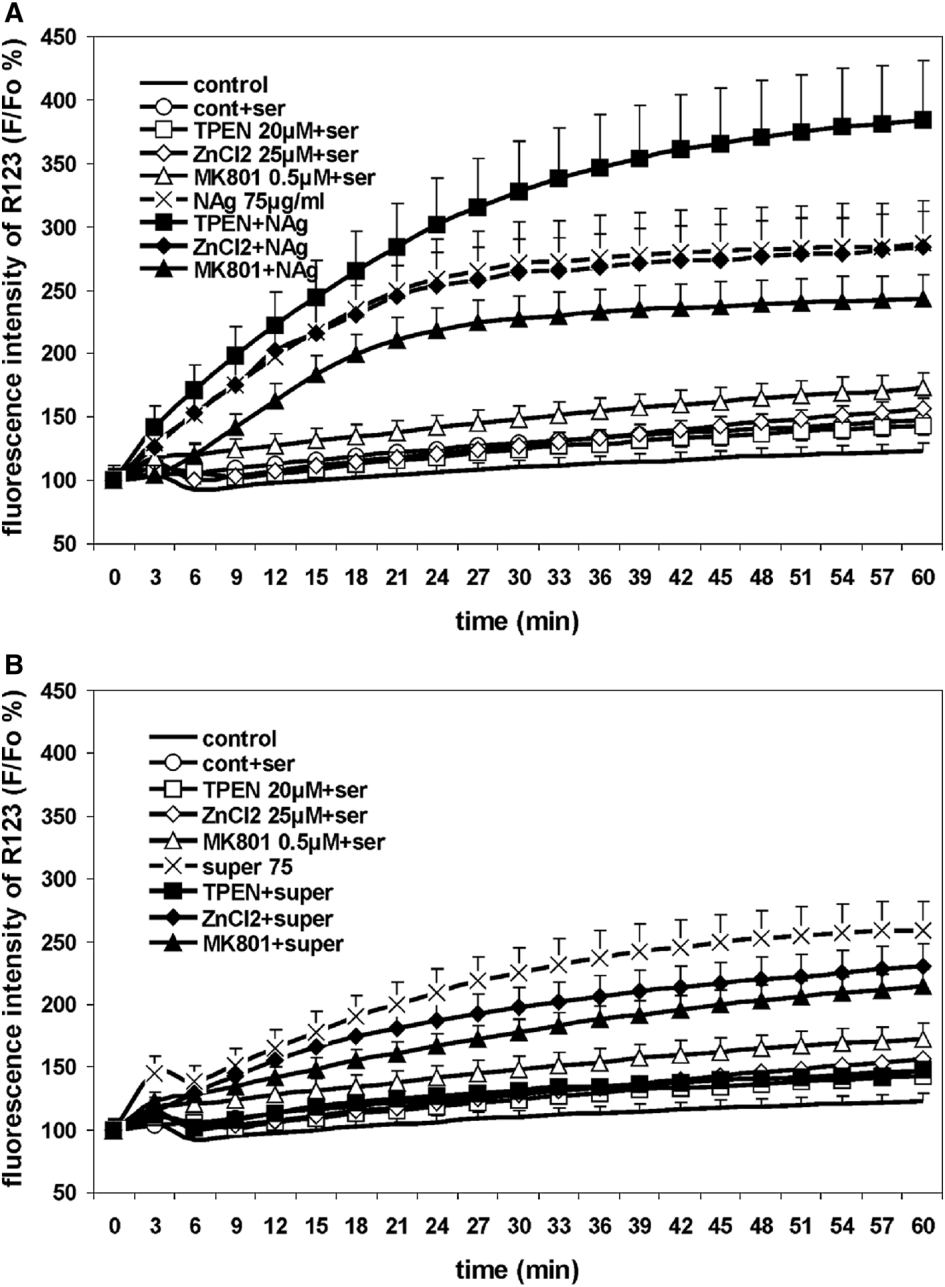
The effect of TPEN, ZnCl_2_, and MK-801 on changes in the mitochondrial membrane potential in primary cultures of rat CGCs exposed to 75 µg/mL nanosilver (NAg) (A) or ionic silver (super 75). The basal fluorescence of cells loaded with rhodamine 123 (R123) was measured after 60 s. Increases in R123 fluorescence reflecting a reduction in the mitochondrial membrane potential are expressed relative to the basal level (R123 F/Fo) as a percentage. The results are mean ± SD from five independent experiments

Exposure of cells to 75 µg/mL nanosilver for 60 min was found to induce a significant increase in the fluorescence by about 160 % above the control value (Fig. 7a). MK-801 added to nanosilver-treated cultures was found to decrease this effect slightly. Supplementation of the culture medium with zinc did not improve the potential, whereas zinc deficiency (20 μM TPEN) significantly disrupted it.

Interestingly, in the case of Ag^+^, we did not observe such an adverse effect of TPEN on the mitochondrial potential, while addition of ZnCl_2_ or MK-801 was found to stabilize the mitochondrial potential and provide a partial protective effect (Fig. 7b).

Production of ROS was determined by measuring the fluorescence intensity of DCF dye after incubation of CGCs for 30 min in the presence of 75 µg/mL NAg or Ag^+^.

**Fig. 8:**
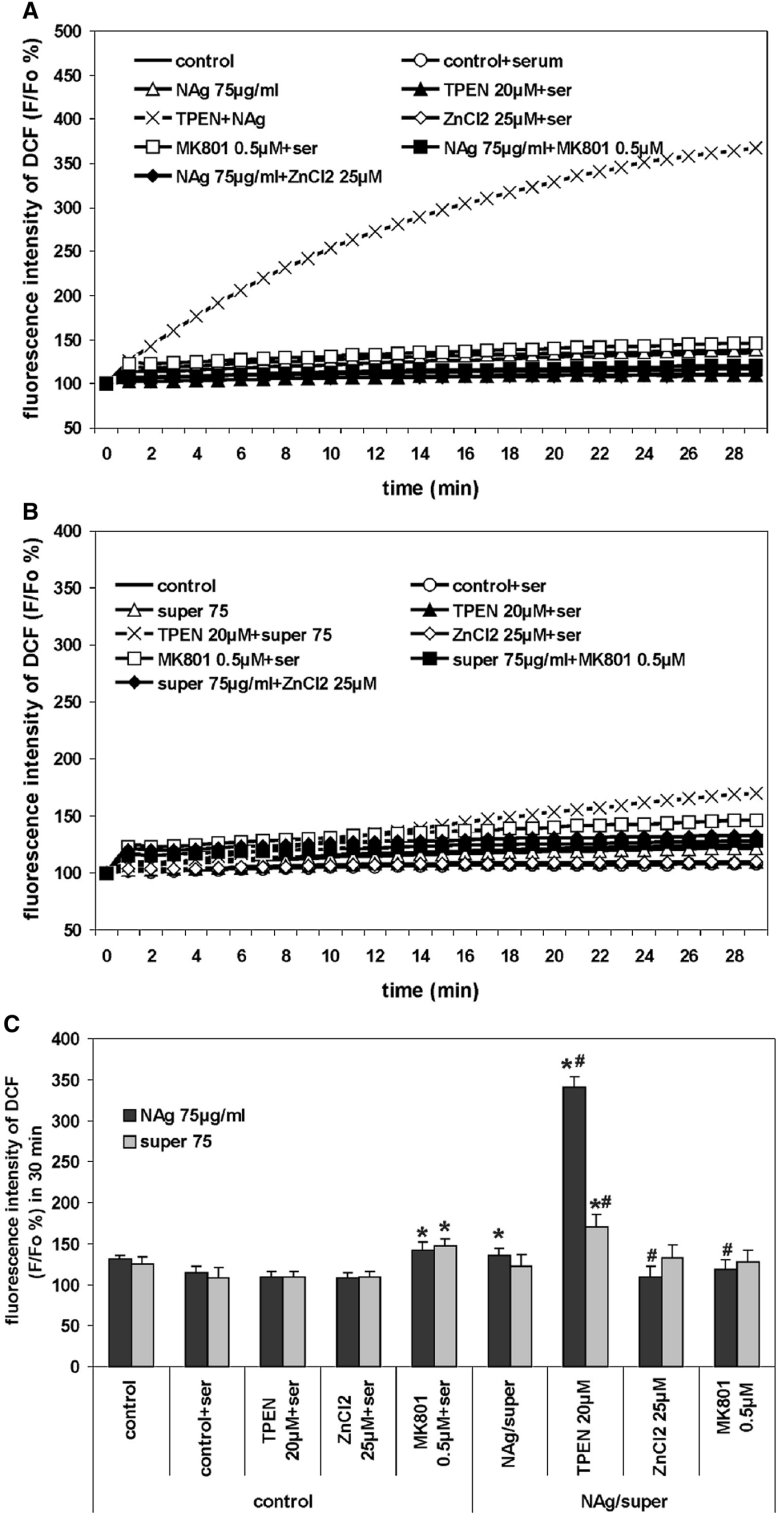
The effect of TPEN, ZnCl_2_, and MK-801 on generation of reactive oxygen species (ROS) in CGCs cultured in the presence of 75 µg/mL of nanosilver (NAg) (**a**) or ionic silver (super 75) (**b**). ROS production was monitored using the fluorescent probe DCF. The basal DCF fluorescence of cells loaded with DCF was measured after 5 min. Increases in DCF fluorescence indicating an enhanced rate of ROS production are expressed relative to the basal level (DCF F/Fo) as a percentage. **c** Generation of ROS at the 30-min time point of the experiment. The results are mean ± SD from five independent experiments. *P < 0.05 versus control untreated cells. ^#^ *P* < 0.05 versus NAg/super 75-treated cells

The fluorescence intensity increased significantly when TPEN was added to nanosilver-exposed cells (by about 250 %) (Fig. 8a) and moderately in Ag^+^-exposed cells (by about 70 %) (Fig. 8b).

ROS production increased significantly relative to the control after a 30-min incubation with NAg but not with Ag^+^ (Fig. 8c). The protective effect provided by MK-801 and ZnCl_2_ was observed only in nanosilver-treated cells and was found to decrease ROS level by about 40 % (*P < 0.05 vs. NAg).

## Discussion

We previously assumed that nanosilver exerts its toxicity in cultured neurons by increasing the rate of the calcium entry through the channels associated with NMDA receptors (Ziemińska et al. 2014). Apart from NMDAR-related destabilization of calcium homeostasis, we also observed a progressive increase in cytosolic zinc levels in neurons under conditions of nanosilver toxicity. The contribution of zinc to the total fluorescence signal was found to be dependent upon the concentration of nanosilver. It is known that increased levels of cytosolic zinc enhance plasma membrane permeability and contribute to degenerative signaling (Medvedeva et al. 2009). The hypothesis has arisen that apart from activation of NMDAR-mediated processes, exposure to nanosilver also leads to disruption of homeostasis of intracellular zinc ions.

Thus, the objective of our present study was to investigate the neurotoxic mechanisms of silver nanoparticles in cultured rat cerebellar granule cells with particular reference to the role of zinc in nanosilver-induced biochemical changes. In order to eliminate intracellular zinc, we used a selective water-soluble and cell membrane-permeable chelator TPEN. In addition, ZnCl_2_ over a range of different concentrations (10–50 µM) was used to supplement cell cultures with zinc. In our experiments, we investigated cerebellar granule neurons which are glutamate- and aspartate-receptive glutamatergic cells (Levi et al. 1991) and possess functional NMDA receptors.

The toxicity of nanoparticles depends on their concentration, size, and surface coating, as well as on the type of cells being investigated (Carlson et al. 2008; Cronholm et al. 2013). In the present study, we used nanoparticles which are coated with 0.2 % PVP to prevent agglomeration. Prior to the addition to the cultures, nanoparticles should be evenly dispersed. Since sonication in water did not result in a stable dispersed form, we therefore selected serum as a medium for sonication (Park et al. 2010). In serum, nanoparticles sediment very slowly and this characteristic provides reproducibility during addition to the cultures. Moreover, it is expected to more closely replicate in vivo conditions where nanoparticles encounter blood serum proteins. However, we also characterize nanoparticles in culture media. Dispersion of nanoparticles in serum stock solution and in culture media was verified by transmission electron microscopy. As indicated in Table 1, we used nanoparticles ranging in size from 5 to 35 nm. However, most of the particles were between 5 and 25 nm and are thus considered in the mid-range with respect to size and toxic potential (Carlson et al. 2008). The degree of dispersion in serum was comparable to that in both biological media. Our results are consistent with previous reports showing that PVP-coated silver nanoparticles do not agglomerate in the presence of 10 % fetal calf serum proteins. Moreover, in protein-free biological media, the presence of thiol-containing amino acids and anaerobic conditions primarily influence the dissolution of silver nanoparticles (Ahlberg et al. 2014).

### Changes Observed in Nanosilver-Exposed CGCs

**Fig. 9:**
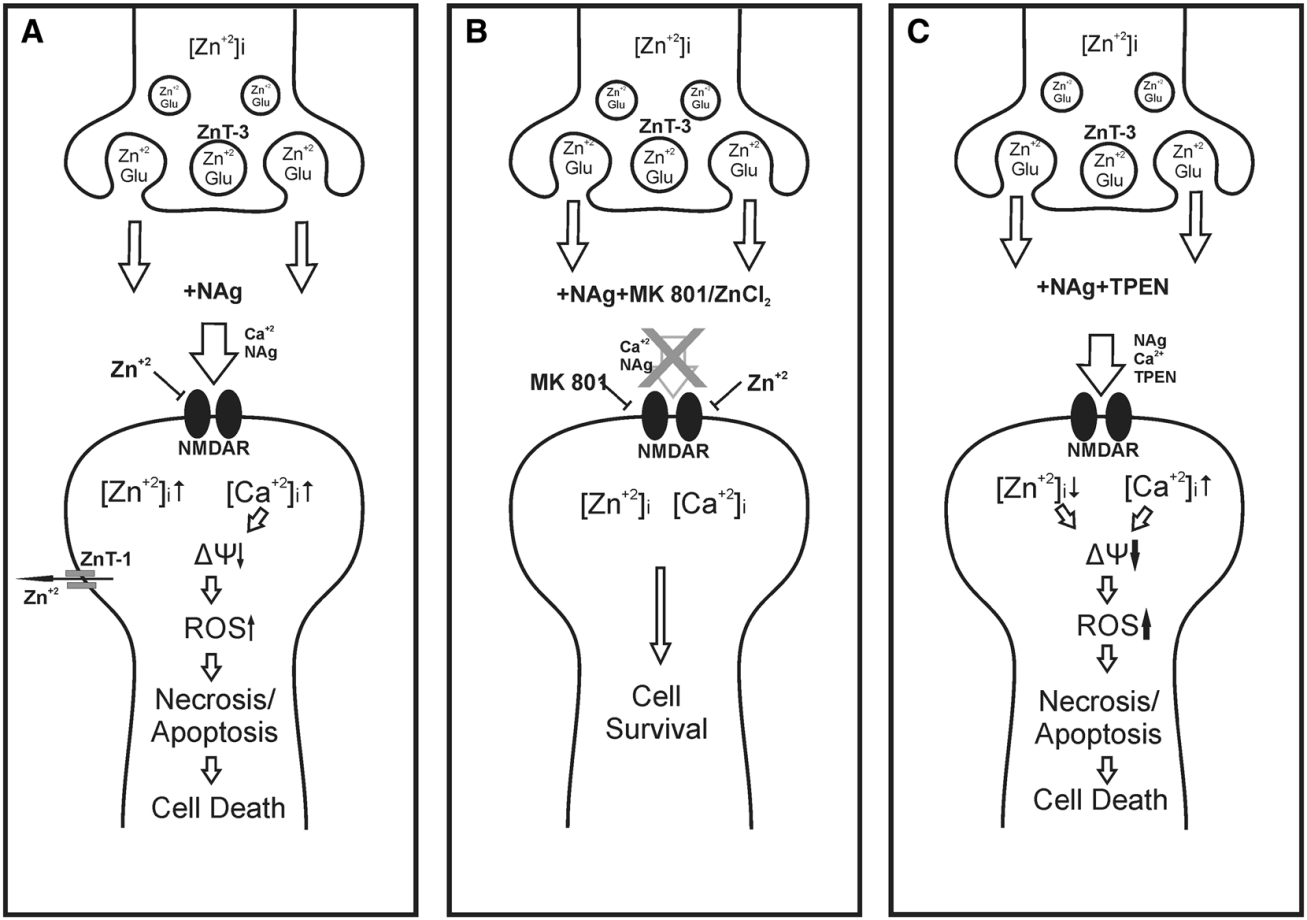
Schematic representation of NAg-induced changes in CGCs **a** under normal conditions **b** during Zn supplementation and **c** zinc depletion by TPEN

Based on the increased intracellular (FluoZin-3AM; Fig. 1a, b and Fluo-3; Fig. 6) and decreased total zinc-derived fluorescence (Fig. 1c), it can be seen that addition of nanosilver influences cellular zinc homeostasis in CGCs. Results of experiments with FluoZin-3AM show instant sevenfold increase in cytosolic Zn-derived fluorescence intensity after addition of NAg within max. 15 min. During the time of experiment, a weak fluorescence appeared also outside the cells. Based on the high selectivity of the dye to free [Zn^2+^], we claimed that cytosolic pool of free zinc increases rapidly after NAg treatment and is subsequently partially disposed out of the cell. This correlates well with drop of total zinc (cytoplasmic free + protein-bound) measured in cell lysates with FluoZin-1 after 30 min (Fig. 1c). Although the precise mechanisms of NAg-induced imbalance between intra/extracellular zinc levels are not known, we propose schematic explanation (Fig. 9). It appears that destabilization of cellular zinc equilibrium is partially a result of enhanced release of zinc from the synaptic pool as a consequence of NMDAR overactivation (Fig. 9a). In CGCs, which express many functional NMDARs, zinc is co-localized with the excitatory neurotransmitter glutamate (Frederickson 1989) and may be synaptically released upon cell activation. Alternatively, enhanced efflux of zinc may be due to the activity of the plasma membrane transporter ZnT-1 which exports zinc out of the cell (Palmiter and Findley 1995) and thus may contribute to critical zinc homeostasis after exposure under depolarizing conditions (Kim et al. 2000). Since we concurrently observed an increase in intracellular zinc in nanosilver-exposed cells, the overactivation of ZnT-1 might counteract the increased levels of cytosolic zinc. Such a protective mechanism for maintaining intracellular zinc was found to be activated following ischemia-induced toxic zinc influx (Tsuda et al. 1997). However, further studies are needed to confirm such a hypothesis. Depletion of synaptic pool of zinc together with selective accumulation of endogenous zinc in damaged neurons was previously described in ischemia, trauma, and following seizures, leading to the “translocation hypothesis”, in which presynaptic zinc is first released extracellularly and then enters postsynaptic neurons mainly through AMPA receptors (Paoletti et al. 2009).

In nanosilver-treated cells, the fluorescence of fluo3-AM was found to increase significantly. Changes in the fluorescence signal of this probe often reflects the simultaneously occurring changes in the concentration of calcium and zinc (Dineley 2007). It is evident that there is an increased cytoplasmic level of “free” zinc. Previously, we confirmed that the total fluo-3 fluorescence signal recorded in neuronal cytosol after exposure to 75 µg/mL nanosilver is mixed a calcium-zinc signal (Ziemińska et al. 2014) and determined the extent to which the detected signals are a result of zinc interference. The results of the current studies using FluoZin-3AM clearly confirm NAg-induced enhancement of cytoplasmic zinc level. Such an increase may represent release of zinc from the protein-bound pool or enhanced cellular entry. Glutamate receptor-induced mobilization of intracellular [Zn^2+^] was proposed to be a general response to glutamate-induced oxidative stress by the mechanism dependent on Ca^2+^ entry (Dineley et al. 2008). Assuming that nanosilver induces overactivation of glutamatergic receptors in CGCs, elevation of extracellular glutamate may be expected. It has been shown that glutamate promotes zinc release from intracellular stores (Dineley et al. 2008) and glutamate-mediated zinc uptake is primarily dependent on voltage-gated calcium channels (VGCCs) and AMPARs (Kerchner et al. 2000; Morris and Levenson 2012).

Increased uptake of radioactive calcium, elevation of intracellular calcium, decreased mitochondrial membrane potential, increased ROS production, and decreased cell viability were additional observations made in CGCs under conditions of nanosilver toxicity (Fig. 9a). These findings are consistent with previous observations. The 10-min exposure to silver nanoparticles in concentration of 75 µg/mL was shown to intensify the entry of radioactive calcium into CGCs which even exceeded the effect evoked by 100 µM glutamate (Ziemińska et al. 2014). Decreased viability of CGCs after NAg treatment was coupled to oxidative stress leading to increased intracellular calcium levels and apoptotic cell death (Yin et al. 2013).

It has been accepted that cellular mitochondria are targets for both calcium and zinc ions, where zinc may be transported through a calcium uniporter (Gazaryan et al. 2007). Silver nanoparticles also have been shown to produce disturbances in mitochondrial function (Costa et al. 2010). Our results which indicate significant loss of mitochondrial potential in nanosilver-treated cells with increased production of ROS are in agreement with the existing data. While investigating the contribution of calcium to the observed effects using MK-801 (an antagonist of NMDAR), we noted an improvement in the mitochondrial membrane potential and lowering of the level of ROS. These findings confirm that nanosilver-induced NMDAR-mediated disturbances in intracellular calcium homeostasis lead to mitochondrial dysfunction and generation of ROS. Whereas relatively low levels of calcium cause rapid, complete, and irreversible loss of mitochondrial membrane potential, zinc was reported to depolarize mitochondria only at relatively high concentrations via a slower, partial, and reversible manner in certain cases (Devinney et al. 2009). Thus, we suggest that the toxic effects exerted by nanosilver on cellular mitochondria are largely mediated by calcium ions.

### Influence of Extracellular Zinc on Nanosilver-Induced Changes in CGCs

Zinc added in a concentration range of 10–50 µM was found to significantly enhance cell viability in nanosilver-treated cultures in a manner similar to that observed upon addition of MK-801. Moreover, even though insignificantly, but added together MK-801 and ZnCl_2_ increased cell viability. This indicates the involvement of an NMDAR-related mechanism. Simultaneously, the lack of positive effect of CNQX on cell viability excluded the contribution of other ionotropic glutamate receptors, like AMPA/KA receptors, to NAg-induced cell death. Indeed, unlike NMDARs, activation of these receptors generally results in very limited mitochondrial calcium loading and toxicity in CGCs (Rego et al. 2001). Thus, we assume that an “excitatory” effect of NAg selectively concerns NMDARs. Despite the fact that AMPA/KA receptors may contribute to the significant elevation of cytosolic zinc, cell toxicity is mainly governed by calcium. AMPARs are also less sensitive to zinc than NMDARs, being inhibited by high milimolar concentrations (Paoletti et al. 2009).

We suspect that zinc may act as a blocker of the NMDA receptor channel to prevent excessive calcium influx into the cell under conditions of receptor activation in the presence of nanosilver (Fig. 9b). Indeed, the receptor blocking effect is also clearly visible during the course of measuring radioactive calcium uptake. All concentration levels of zinc reduce both nanosilver- and glutamate-induced (agonist of NMDAR) calcium influx to CGCs. It has been demonstrated that zinc may block NMDAR currents at micromolar concentrations (Westbrook and Mayer 1987) and attenuate calcium influx through the receptor channel (Eimerl and Schramm 1993). Moreover, zinc was previously reported to inhibit acute glutamate toxicity in CGCs (Eimerl and Schramm 1991) and prevent apoptosis in a keratinocyte cell line (Parat et al. 1997).

Since NMDAR complex constitutes an ion channel selective not only for Ca^2+^ but also for Na^+^, K^+^, modulatory effect of zinc may also concern the intra/extracellular equilibrium of these cations. It is of importance because increasing intracellular Na^+^ and Ca^2+^ concentrations decrease or even reverse activity of Na^+^/Zn^2+^ and Ca^2+/^Zn^2+^ exchangers (Qin et al. 2008). Elevated zinc was shown not to compromise Na^+^/K^+^ homeostasis while acting through voltage-dependent calcium channels (VDCC) (Pivovarova et al. 2014). Modulation of NMDAR-activated changes in cations homeostasis by zinc, especially under conditions of NAg exposure, need further study.

We observe that added zinc also decreased significantly, but not completely, ROS production after treatment with nanosilver but it was found to be ineffective with respect to improving the mitochondrial potential. Zinc has been described as being involved in cellular mechanisms of redox potential protecting thiol groups of essential proteins, and it is known that it is an essential cofactor of the stabilizing antioxidative enzyme SOD-1 (Bray and Bettger 1990). When added to the cell culture, zinc exerts a protective effect through its antioxidant activity (Richard et al. 1993).

When released from synaptic vesicles, zinc reaches an extra-synaptic concentration in the hundred micromolar range (Assaf and Chung 1984). During enhanced activity of NMDA receptors which we postulate takes place with nanosilver treatment of CGCs, even higher levels of [Zn]_ex_ are possible. We expected that additional supplementation with 10–50 micromolar ZnCl_2_ would enhance nanosilver toxicity by an adjective toxic effect. The neurotoxic effect of exogenously applied zinc at a concentration of 40 µM upon prolonged exposure (up to 24 h) was demonstrated in vitro (Sheline et al. 2000), whereas shorter exposures were found to be cytotoxic with respect to CGCs at higher concentrations ranges 100–500 µM (Manev et al. 1997). The possibility exists, that apart from enhancing NMDAR activity, nanosilver may also interact in an inhibitory manner with other types of CGC receptor/channels which provide routes for entry of zinc into the cell, thereby reducing inward transmission of zinc ions. AMPA/kainate glutamate receptors, voltage-gated calcium channels, and the Na/Zn antiporter are the main candidates (for a review see: Morris and Levenson 2012). It is also likely that toxic elevation of [Zn]_in_ is prevented by the same cellular mechanisms responsible for zinc homeostasis i.e., transporters and/or binding to metallothioneins. The ZnT-1 transporter was shown to be up-regulated during ischemia-induced toxic zinc influx (Tsuda et al. 1997), and cells overexpressing it are provided with resistance to zinc-induced death (Palmiter and Findley 1995). On the other hand, as we stated above, enhanced release of glutamate/zinc from the synaptic pool might overactivate ZnT-3 which is responsible for transporting zinc to the synaptic vesicles and thus lowering the cytoplasmic level of zinc.

### Influence of Zinc Deficiency on Nanosilver-Induced Changes in CGC (TPEN Effect)

It was demonstrated that administration of the cell membrane-permeable zinc chelator TPEN reduces zinc-induced death of cortical neurons (Canzoniero et al. 2003). This indicates that the chelator eliminates the toxic consequences of excessive zinc by restoring its cellular homeostasis.

In our experiments, elimination of zinc by TPEN led to the complete death of CGCs exposed to 75 µg/mL nanosilver. Moreover, concurrent exposure of cells to the low concentrations of nanosilver (2.5–10 µg/mL) and TPEN induced characteristic apoptotic changes in the cell nuclei, although added alone TPEN did not exert such effect (Fig. 3). It has been shown that zinc deprivation induced by addition of TPEN may lead to DNA fragmentation and trigger apoptosis (Parat et al. 1997); however, at the concentration levels used in our experiments, it evoked cell death only in the presence of nanosilver. Application of TPEN also aggravates the mitochondrial membrane and generates large amounts of free radicals (Fig. 9c). The observed toxic effects of TPEN are presumably triggered by excessive chelation of [Zn^2+^]_in_ which is needed for cellular processes with subsequent loss of its antioxidant properties. The involvement of zinc in cellular redox mechanisms occurs by binding to a great number of biological molecules (Bray and Bettger 1990). Furthermore, chelation of synaptically derived zinc by TPEN abolishes its inhibitory effect on NMDAR, thereby enhancing NMDAR-mediated calcium uptake (Fig. 3), ROS production (Fig. 8c), and disturbing mitochondrial potential (Fig. 7). The toxic effect of TPEN observed in nanosilver-treated cells reflects pathological lowering of intracellular zinc and its availability for use as cofactors of enzymes and transcription factors.

On the other hand, the same concentrations of TPEN were found to be protective in certain experiments. We observed lowering of the intracellular calcium level in nanosilver-treated cells subjected simultaneously to TPEN (Fig. 6). This clearly indicates significant participation of a zinc-derived component in cytosolic Ca^2+^/Zn^2+^ fluorescence. The increased level of cytosolic zinc under nanosilver exposure is presumably connected to the overactivation of NMDAR as concluded from the reversing effect of MK-801 and ZnCl_2_. Thus, it is possible that nanosilver particles cause immediate disturbance of zinc homeostasis but to a low and variable extent. Possibly, a nanosilver-induced increase in cytosolic zinc measured in the experiment with fluo-3 (Fig. 6) is not high enough to induce adverse effects inside the cell.

### Comparing Nanosilver and Ag^+^ Effects on CGCs

Previous reports have discussed whether the mechanisms of silver toxicity depend on its formulation (for a review see: Hadrup and Lam 2014) and whether nanosilver interacts specifically with biological systems. It is widely accepted that Ag^+^ is liberated from the surface of particles and may, to some extent, be responsible for the toxic effects of nanosilver. The present results and our previous report (Ziemińska et al. 2014) indicate that NMDAR-mediated toxicity is a mechanism specific for nanoparticulate but not ionic silver. Based on our previous paper (Ziemińska et al. 2014), supernatant obtained after centrifugation of a nanosilver stock solution was used as a control to recognize the component of the action resulting from Ag^+^ liberated from the nanoparticles.

While comparing the effects of nanosilver and Ag^+^ on CGCs, it is clear that “nano” formulation of silver more effectively interacts with the investigated processes. Enhancement of calcium uptake, increased intracellular Ca^2+/^Zn^2+^ levels, and ROS production are largely nanosilver-triggered events. Our results reveal that nanosilver contributes to greater extent than Ag^+^ to cellular toxicity. This is consistent with other reports (Yin et al. 2013).

Effective protection provided by MK-801 is observed in most of the experiments in nanosilver- but not Ag^+^-treated cultures. This confirms the involvement of NMDAR in the pathological changes in CGCs as a nanosilver-specific mechanism. In contrast to nanosilver-treated cells, long lasting (24 h) but not short-time experiments (30–60 min) revealed positive effect of NMDAR antagonist. It is possible that NMDAR-mediated mechanisms are switched on during prolonged time of incubation with Ag^+^ as secondary mechanisms. Likewise, zinc homeostasis was altered in nanosilver-exposed cells. This represents the most pronounced effect of zinc supplementation and intensified TPEN toxicity.

## Conclusions

The results of this study stated the role of zinc in nanosilver-evoked CGC death. It is evident that exposure to increased concentrations of nanosilver leads to an imbalance between extra/intracellular zinc levels. The same does not hold true for exposure to Ag^+^. Excessive increase in cytosolic level of zinc with its subsequent release from glutamatergic synapses are related to the contribution of zinc in processes mediated by overactivation of NMDARs, specifically under nanosilver exposure. However, the results indicate, in contrast to our expectations, that zinc homeostasis, although altered during exposure to nanosilver, does not contribute significantly to cell death. This conclusion is supported by the observation that the zinc chelator TPEN does not eliminate toxic changes evoked by nanosilver but instead tends to increase them leading to cell death. In contrast, zinc supplementation exerts a positive effect, which is likely due to inhibiting the NMDA-sensitive glutamate-gated calcium current.

## Abbreviations

AMPA/KA receptors: Glutamatergic AMPA and kainate receptors
BME: Basal Eagle’s medium
CGC: Cerebellar granule cells
CNS: Central nervous system
DCF: 2′,7′-Dichlorofluoresce in diacetate
EtHD: Ethidium homodimer
FCS: Fetal calf serum
MK-801: Noncompetitive antagonist of NMDA receptor
NAg: Silver nanoparticles
NMDAR: Glutamatergic *N*-methyl-d-aspartate receptor
PVP: Polyvinylpyrrolidone
R123: Rhodamine123
ROS: Reactive oxygen species
TEM: Transmission electron microscopy
TPEN: *N*,*N*,*N*,*N*-tetrakis(2-pyridylmethyl)ethylenediamine
Zn-T1, Zn-T2, Zn-T3: Transmembrane zinc transporters
